# Atypical Management of Stroke Caused by Mucormycosis: Case Report and Review of the Literature

**DOI:** 10.2147/IMCRJ.S419609

**Published:** 2023-08-23

**Authors:** Lina Okar, Boulenouar Mesraoua, Dirk Deleu

**Affiliations:** 1 Department of Medical Education, Hamad Medical Corporation, Doha, Qatar; 2 Department of Neurology, Hamad Medical Corporation, Doha, Qatar

**Keywords:** mucormycosis, stroke, thrombolysis

## Abstract

Mucormycosis is an opportunistic infection that affects immunocompromised patients, especially those with uncontrolled diabetes. Clinical presentation depends on the site of infection. Complications arise when the pathogen invades the host tissue causing vascular necrosisand distortion. Disease course is fast, and most of the time it has a poor or fatal outcome. Rhino-orbito-cerebral mucormycosis is the most common presenting form. Although initial complaints include fever, sinusitis, nasal discharge, headache, facial pain, and swelling, it should be kept in mind that patients might present during the complication period with a hemiparesis or altered mental status. Here, we present a case of patient who presented with a stroke and further workup revealed the presence of mucormycosis. According to our knowledge, this is the first case of mucormycosis complicated with stroke that was managed with thrombolysis.

## Introduction

Mucormycosis is an aggressive, destructive, and opportunistic fungal infection. As an infection that happens almost exclusively in immunocompromised patients, those with diabetes are at a great risk.^1^ Rhino-orbital-cerebral mucormycosis is the most common presentation with a percentage of 60%. Presentation is usually with ophthalmological complaints and facial swelling. Early recognition and treatment are the cornerstone in improving prognosis. Patients who have a cerebral complication are considered to have poor prognosis and high fatal outcome.^2^ Brain damage in mucormycosis infections is diverse and primarily due to the direct spread of infection which distorts brain tissue and its vasculature through occlusion, dissection or narrowing of arteries, resulting in hemorrhagic or ischemic stroke.^3^ Although cerebral complications are common and have been reported before, the management of stroke in this specific context is still not described.^3–5^ It is undoubtful that early recognition of the pathogen and directed antifungal treatment is essential in improving outcome in the case of mucormycosis. Furthermore, patients who present with cerebral involvement such as stroke caused by mucormycotic infection also require early recognition, but how other interventions like thrombolysis might affect the outcome is not described in the literature.

## Case Presentation

A 26-year-old man with uncontrolled diabetes was brought to the Emergency Department (ED) at Hamad General Hospital due to sudden onset of left-sided weakness associated with drowsiness, one hour prior to arrival. Before this admission, he had a one-week history of headache and fever. Three days before, he developed right-sided facial swelling and pain associated with right-sided blurring of vision for which he had been prescribed an oral antibiotic. In the ED, he was still complaining of left-sided weakness that started to improve gradually.

On examination, the patient was drowsy. His blood pressure was 122/81 mmHg and pulse rate was 92 per minute and regular, and he was afebrile (36.6°C). He was able to open his eyes to verbal stimuli, answer questions, perform two tasks correctly, and move the right side of his body spontaneously. Glasgow Coma Scale was 14. Speech was mildly dysarthric. There was right-sided diffuse facial swelling extending from the right upper eyelid down to the right side of the upper lip. He was not able to open his right eye fully. Visual acuity of the right eye was limited to light perception. The right pupil was 5 mm dilated and non-reactive. The visual acuity of the left eye was normal and visual fields were intact. The left pupil was normal and reactive to light. Horizontal and vertical eye movements were normal bilaterally. Fundoscopy revealed features of right central retinal vein thrombosis. Facial sensation was intact for all modalities. There was a mild left-sided upper motor neuron facial palsy. The rest of the cranial nerve examination was normal. Motor exam revealed left-sided weakness of both upper and lower limbs (power 2/5 and 4/5, respectively). The left upper and lower limbs were flaccid, and reflexes were sluggish in the presence of bilateral Babinski sign. Pin prick sensation was present bilaterally with right parietal extinction phenomenon on bilateral tactile stimulation. He had no ataxia. His National Institute of Health Stroke Scale (NIHSS) was 9.

In the presence of a suspicion of a facial infection on the right side and left-sided hemi-deficit, the diagnosis is expected to be related to this infection. Hence, the initial differential diagnosis included ischemic stroke due to either arterial or venous infarction, intracranial hemorrhage, or seizures with post-ictal weakness.

**Figure 1:**
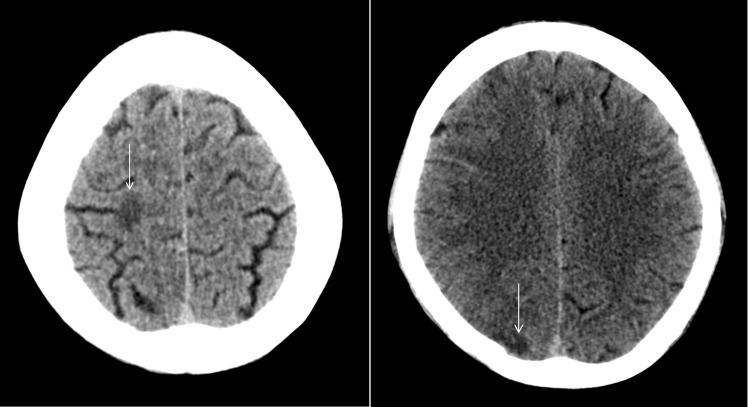
The Left part is showing axial CT scan of the brain which shows right parietal subcortical white matter hypodensity (arrow). Right: Fig Axial CT scan of the brain shows right frontal subcortical white matter hypodensity (arrow).

**Figure 2:**
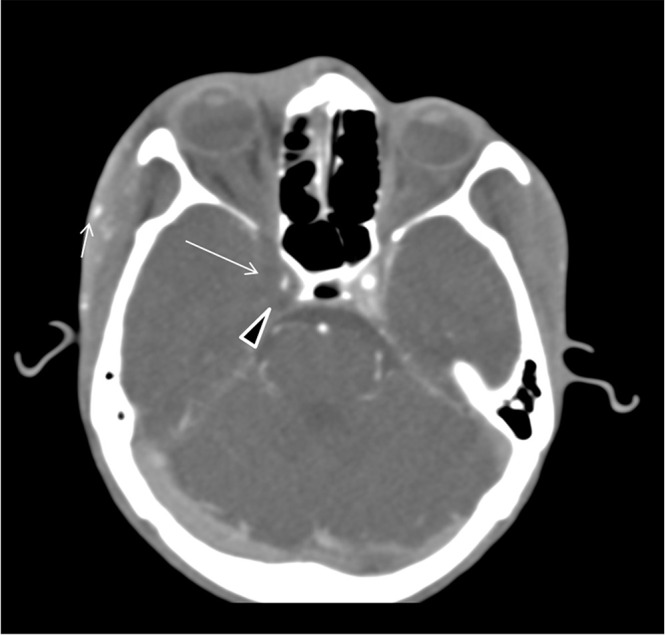
Axial post contrast CT angiography of the brain shows symmetrical non-enhancing right cavernous sinus (arrowhead), reduced caliber of the right internal carotid artery cavernous segment (arrow) swelling of the right temporal lobe and swelling and enhancement of the subcutaneous right temporal region as well as the orbital septum (short arrow).

At this stage, a critical decision to thrombolysis the patient for a possible ischemic stroke is needed. Urgent multimodal brain CT was performed and showed right frontal scalp swelling extending to the eyelids, hypodense areas in the right frontal and parietal subcortical white matter and right thalamus (Figure 1). The CT angiogram of the head, which was unfortunately not optimal because of movement artifacts, showed symmetrical non-enhancing right cavernous sinus, reduced caliber of the cavernous segment of the right internal carotid artery, swelling and enhancement of the subcutaneous right temporal region and orbital septum (Figure 2). Because of these significant additional findings, an urgent MRI diffusion scan was performed and showed multiple areas of diffusion restriction involving the right ventral thalamus, posterior limb of the internal capsule, corona radiata, and cortical/subcortical areas all in the right middle cerebral artery (MCA) distribution indicating an embolic ischemic stroke.

The laboratory results revealed 9.7 × 10^3^/µL white blood count with 8.3 × 10^3^/ µL neutrophils, hemoglobin 14.1 g/dL, platelets 257 × 10^3^/µL, creatinine 70 µmol/L, sodium 131 mmol/L, chloride 95 mmol/L, bicarbonate 24.9 mmol/L, potassium 4.3 mmol/L, and HbA1c 16.4%. Based on the clinical and neuroimaging findings the patient was given intravenous t-PA. Reassessment after thrombolysis showed no change in physical exam and NIHSS remained at 9. The patient was admitted to intensive care unit, and IV antibiotics were started.

Next day, his GCS dropped by 2 units. He developed bilateral ophthalmoplegia. In primary position, the right eye was fixed in central position, and the left eye was adducted and deviated downward. The right pupil remained 5 mm dilated and non-reactive and the left pupil was still reactive. Left upper and lower limb power dropped to 0/5. An urgent brain CT scan showed a large right frontal temporo-parieto-occipital infarct and midline shift of 6 mm. Consequently, he underwent urgent decompressive hemicraniectomy.

**Figure 3:**
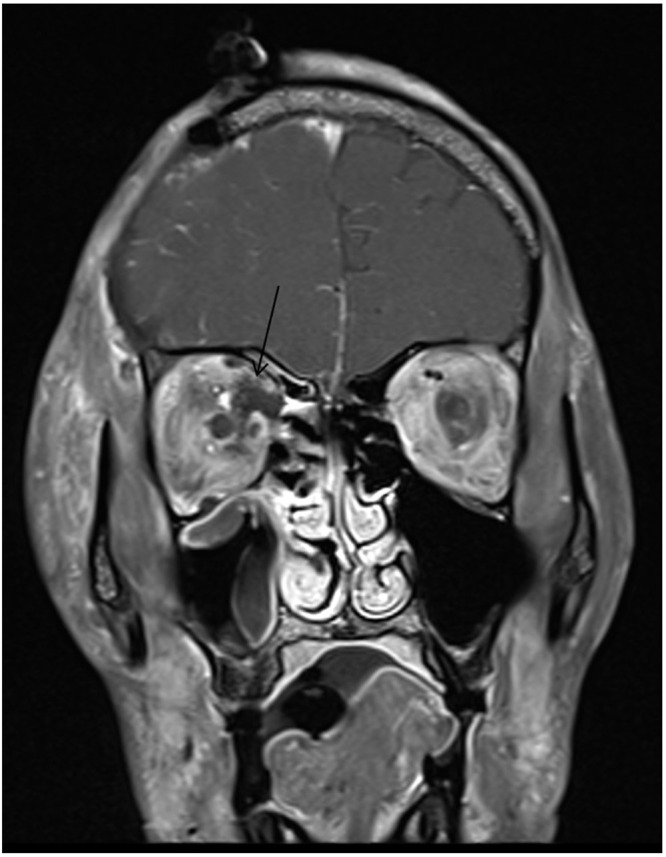
Coronal T1 post contrast shows right intra-orbital collection (arrows).

**Figure 4:**
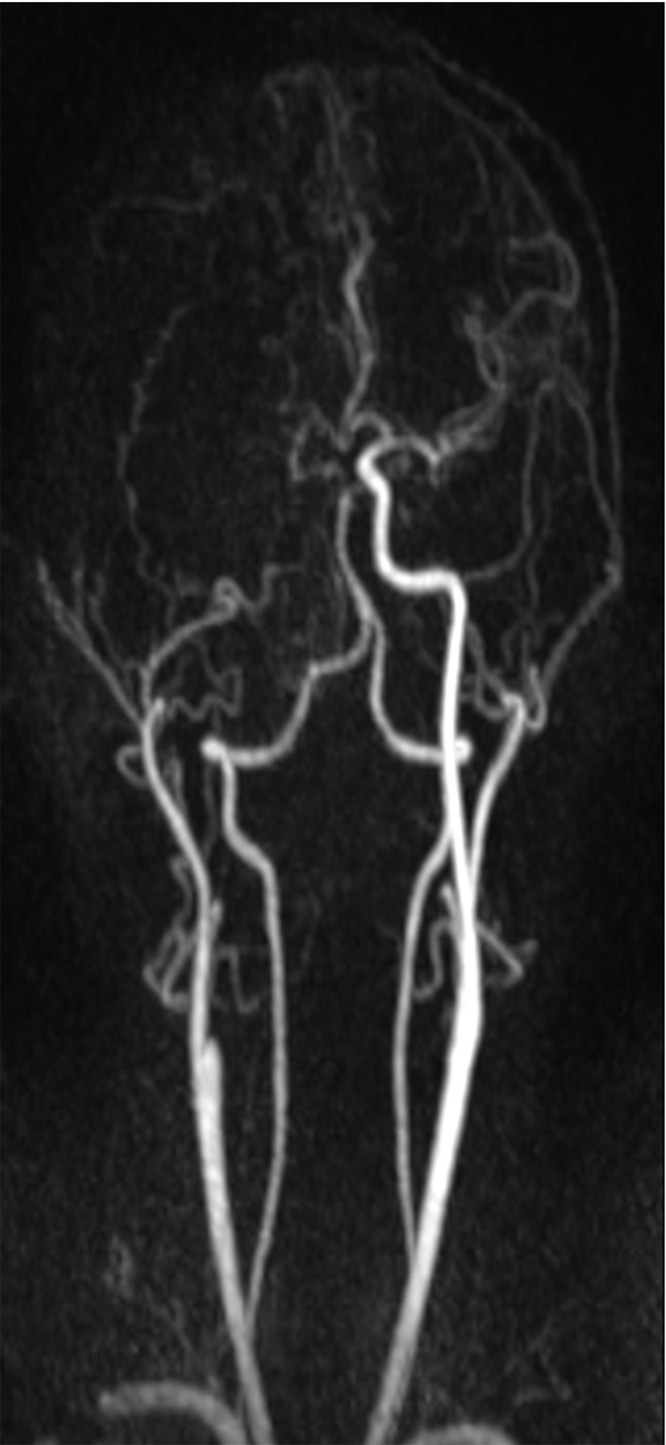
Post processing MRA of the neck shows right internal carotid artery occlusion from the origin.

On day 3, the patient was intubated but not sedated. Physical exam showed GCS of 10, bilateral ophthalmoplegia (as described before), and dense left-sided hemiplegia. MRI of the head and orbit, as well as MRA and MRV, were performed and showed maxillary, frontal, and spheno-ethmoidal sinusitis complicated by right-sided facial and orbital cellulitis, right orbital subperiosteal abscess (Figure 3), right cavernous sinus and ophthalmic vein thrombosis, and occlusion of the right internal carotid artery (Figure 4). In addition to recent right middle cerebral artery territory infarction with microhemorraghic changes, shift of the midline structure and mass effect. Invasive sinus infection in a patient with diabetes mellitus raises the possibility of a fungal sinusitis. Therefore, liposomal amphotericin B was started in addition to vancomycin and ceftriaxone. Nasal sinus biopsy was performed, and culture showed *Rhizopus* species (mucormycosis).

Two weeks after the stroke he showed gradual improvement in the level of consciousness. A tracheostomy tube was placed. His level of consciousness eventually returned to normal. He was able to follow commands. The right-eye ophthalmoplegia persisted but the left-eye movement became normal. The facial swelling resolved. He continued to have dense left-sided weakness 0/5 but was able to sit with support.

## Discussion/Conclusion

Mucormycosis (phycomycotic or zygomycosis) is an angioinvasive infection caused by the ubiquitous filamentous fungi of the Mucorales order of the class of Zygomycetes.^6^ The three genera responsible for most cases are *Rhizopus, Lichtheimia*, and *Mucor*.^3^ Phycomycetes are commonly found in the environment and can colonize the oral mucosa, nose, paranasal sinuses, and throat.^3^ Human infection occurs through inhalation of phycomycete spores into the respiratory tract. The organism enters through the nonlaryngeal and orbital passages into the intracranial cavity either by direct extension through bone and soft tissue or by invading the vascular channels.^3^

Clinical presentations of mucormycosis infection include rhinocerebral (rhino-orbito-cerebral), pulmonary, cutaneous, gastrointestinal, disseminated, and uncommon presentations.^6^ Predisposing conditions include malignant hematological disease, chronic and severe neutropenia, poorly controlled diabetes mellitus, iron overload, major trauma, chronic use of corticosteroids, illicit intravenous drug use, and malnourishment.^2^ However, the most common predisposing risk factor for mucormycosis is diabetes mellitus (36–88%) with the large majority having diabetic ketoacidosis.^3^,^4^,^7^ Rhino-orbito-cerebral mucormycosis (ROCM) is the commonest form of this disease entity in diabetic patients.^7^

ROCM is a serious life-threatening infection. Mucor is attracted to blood vessels and invasion of their wall, particularly arterial, which is the pathological trademark of the infection.^8^ The disease spreads to the cavernous sinus, internal carotid artery, and ultimately to the brain, which was the case in our patient. Mucormycosis causes pseudoaneurysms, partial thrombosis, narrowing, and arteritic irregularities. It can result in intracranial hemorrhage due to mycotic pseudoaneurysms, arterial dissection, or venous congestion.^7^ ROCM can cause basilar artery territory infarction as well.^4^,^5^,^9^ Diabetic ketoacidosis, local acidosis, and hyperglycemia inhibit the affinity and effectiveness of macrophages, altering host defense mechanisms, and promote growth of mucors.^3^

Typical clinical features of rhino-orbito-cerebral zygomycosis include changes in mental status, fever, headache, epistaxis, sinusitis, partial or total ophthalmoplegia, loss of vision, proptosis, chemosis, periorbital edema, ptosis, granular or purulent nasal discharge, nasal ulceration, eschar in the nasal or oral cavity, and stroke.^7^ Our patient had fever, facial swelling, and pain, as well as loss of vision and ptosis, prior to stroke symptoms.

Up to 50–80% of patients with only invasive sinus disease survive. However, if infection spreads to the brain, the case fatality ratio may exceed 80%.^3^ The prognosis is always very poor once the carotid artery is involved. Thajeb et al reported a series of 6 patients with rhino-orbito-cerebral mucormycosis with large vessel involvement, and all patients eventually died.^4^

It is often difficult to diagnose mucorthrombosis before it causes serious infarctions of the brain. This is because direct histological examination of the intracranial vessels is not practical and in diabetes mellitus it is difficult to differentiate between diabetes arteriopathy and mucorthrombosis.^4^

Mucorthrombosis of the ophthalmic artery, occlusion of the central retinal artery, and direct invasion of the optic nerve can cause acute visual loss.^4^ In our patient, visual loss was most likely - and due to direct invasion of the fungus - caused by central retinal vein thrombosis. Early diagnosis of ROCM is possible when the treating physician is familiar with the clinical presentations of this infection and is suspecting the disease.^4^

Clinical features and imaging can help in the diagnosis, but histopathological diagnosis is the gold standard. Contrast-enhanced CT scans show the edematous mucosa, fluid filling the ethmoid sinuses, and destruction of periorbital tissues and bone margins. Bone destruction is often seen only late in the infection course after soft-tissue necrosis has occurred.^6^ Brain MRI can show the intradural and intracranial extent of ROCM with thrombosis of the cavernous sinus, and cavernous portions of the internal carotid artery which was the case in our patient. Contrast-enhanced MR imaging may also reveal perineural spread of the infection.^6^ MR imaging is more sensitive than CT scan in detecting infection of orbital soft tissue. MRI and CT scans can be normal early in the disease. High-risk patients should always be subjected to surgical exploration with biopsy analysis of the suspected areas of infection.^6^ Imaging studies are non-specific for ROCM and diagnosing ROCM almost always requires histopathological evidence of fungal tissue invasion.^6^

Factors that support favorable outcome include early diagnosis and initiation of antifungal treatment with amphotericin B as well as complete surgical debridement with ocular exenteration.^3^,^4^ There are several case reports of patients who developed strokes related to rhino-orbito-cerebral mucormycosis, but as far as we know this is the first case that has been thrombolysed.

In our patient thrombolytic therapy did not seem to slow the rapid progression of the disease as within 12–24 hours after thrombolysis the patient developed right internal carotid artery occlusion, massive right-sided infarction. On the day of thrombolysis, there were patchy infarcts in the right internal carotid artery distribution, which could be related to mucorthrombosis causing direct invasion and obliteration of large arteries. On the other hand, the contribution of thrombolytic therapy to the long-term prognosis is doubtful.

## Data Sharing Statement

All data related to this case are available in our records by contacting the corresponding author.
